# Psychometric Validation of the Autism Impact Measure (AIM)

**DOI:** 10.1007/s10803-019-04011-2

**Published:** 2019-04-09

**Authors:** Richard Houghton, Brigitta Monz, Kiely Law, Georg Loss, Stephanie Le Scouiller, Frank de Vries, Tom Willgoss

**Affiliations:** 1 Personalized Health Care Data Science, Real World Data, F. Hoffmann-La Roche Ltd, Grenzacherstrasse. 124, 4070 Basel, Switzerland; 2 School CAPHRI, Maastricht University, Maastricht, The Netherlands; 3 Kennedy Krieger Institute, 707 North Broadway, Baltimore, MD 21205 USA; 4 Johns Hopkins University School of Medicine, 733 North Broadway, Baltimore, MD 21205 USA; 5 Patient Centered Outcomes Research, Biometrics, Roche Products, Ltd, Falcon Way, Welwyn Garden City, UK; 6 Department of Clinical Pharmacy & Toxicology, Maastricht UMC+, The Netherlands

**Keywords:** Autism spectrum disorder, Outcome, Treatment, Symptoms, Psychometric validation

## Abstract

The Autism impact measure (AIM) is a caregiver-reported questionnaire assessing autism symptom frequency and impact in children, previously shown to have good test–retest reliability, convergent validity and structural validity. This study extended previous work by exploring the AIM’s ability to discriminate between ‘known-groups’ of children, and estimating thresholds for clinically important responses. Data were collected online and electronically on computer and mobile devices; hence, it was also possible to confirm other psychometric properties of the AIM in this format. This study provides confirmatory and additional psychometric validation of the AIM. The AIM offers a valid, quick and inexpensive method for caregivers to report core symptoms of autism spectrum disorder (ASD) including communication deficits, difficulties with social interactions and repetitive behaviors.

**Electronic supplementary material:**

The online version of this article (10.1007/s10803-019-04011-2) contains supplementary material, which is available to authorized users.

## Introduction

The Autism impact measure (AIM) is a caregiver-reported questionnaire, designed to be used in clinical trials and clinical practice to assess effectiveness of interventions in Autism Spectrum Disorders (ASD; Kanne et al. 2014; Mazurek et al. 2018). It consists of 41 items, and each is rated on a 5-point Likert-type scale for both frequency and impact. Caregiver-reported clinical outcome assessment measures such as the AIM may have some advantages over established interview-administered measures in that they are often less time consuming and do not need specifically trained personnel to administer. As a result, the AIM could also potentially serve as suitable tool for real-world monitoring of ASD symptoms, embedded in more routine care or remote settings. Additional advantages of the AIM are that other commonly used scales have either been created for diagnostic purposes only (e.g. autism diagnostic observational scale: ADOS; Lord et al. 2012), were developed and tested according to older and more narrow definitions of ASD (e.g. Behavioral Summarized Evaluation Scale: BSE; Barthélémy et al. 1997 and Real Life Rating Scale: RLRS; Freeman et al. 1986) or focus on non-core or not all core characteristics (e.g. Social responsiveness scale: SRS-2; Constantino and Gruber 2012). The AIM, in contrast, has been shown to exhibit 5 “theoretically and empirically meaningful” symptom domains, namely; Repetitive Behavior, Communication, Atypical Behavior, Social Reciprocity and Peer Interaction (Mazurek et al. 2018). The domain scores utilize only 29 of the 41 items, while the total score still builds on all items. Higher domain and total scores represent worse severity of ASD symptoms.

While the AIM has shown to have good test–retest reliability, cross-informant reliability and convergent validity with other scales (Kanne et al. 2014; Mazurek et al. 2018), other important validation questions remain untested. Importantly, the ability of the AIM to detect differences between known subgroups of individuals with ASD has not been demonstrated. Known-group analysis is needed to demonstrate that a measure is sensitive and able to discriminate between subgroups previously established to have differences in severity. Furthermore, there has been no attempt to estimate magnitudes of such differences that constitute clinically meaningful changes. Successful validation of these two concepts is fundamental for confidence to use the AIM in any study wishing to demonstrate efficacy of a given intervention. Therefore, the primary objective of our study was to address these gaps in a large and representative sample. Also, because participants in our study completed the questionnaire electronically, rather than on paper, secondary objectives were to assess the time needed to complete the AIM and confirm other measures of psychometric validity in this format, including internal/external validity and confirmatory factor analysis.

## Methods

### Data Collection

Participants were invited to take part in our study via the Simons Foundation Powering Autism Research for Knowledge (SPARK) cohort. SPARK is an online community for people with ASD and their families in the United States (US), who are interested in participating in ASD research (SPARK Consortium 2018). Families complete a battery of questionnaires on entry to the cohort, and third-party researchers (industry or academic) can recruit the same families to their studies thereafter. All data generated are anonymized and made linkable via unique identifiers. To be eligible for the current study, participants had to be the main caregiver living in the same household as a child with ASD, and were instructed to answer the AIM in relation to only the oldest child with ASD between 3–17 years. All data used for the study were provided by caregiver-report and were collected during September and October 2017 as part of a wider study on non-drug treatments and potential barriers to care. Details on recruitment and data collection have been published elsewhere (Monz et al. 2019).

### Analysis Populations

In total, 5001 participants returned the AIM survey. As the current AIM scoring system does not mention methods for handling missing data, our main analysis population of interest was those 4415 participants who completed all items. We qualitatively checked for differences in characteristics of those who did not complete all items however, as well as between those who took part on either a vertical or horizontal layout. In the horizontal layout, possible responses to each item were displayed ‘across’ the screen. In the vertical layout the possible responses were displayed ‘down’ the screen (i.e. underneath one another). The format deployed was based on the screen dimensions of the device used to complete the survey (e.g. vertical layout for most mobile phones and horizontal layout for laptops/computers).

A subgroup of respondents had also previously completed the Social communication questionnaire-lifetime (SCQ; Chandler et al. 2007; Rutter et al. 2003) and/or the Repetitive behaviors sale-revised (RBS-R; Mirenda et al. 2010) as part of the SPARK procedures. We linked this data for convergent validity analysis so long as the age of the child differed by no more than 1 year between the time of AIM assessment and the time of SCQ/RBS-R (exact date of SCQ/RBS-R was unknown). Linked sample sizes available were 3064 for the SCQ and 3190 for the RBS-R. There was a significant overlap of 2571 participants who completed all of AIM, SCQ and RBS-R.

### Analysis Plan

#### Descriptive Analysis

We calculated the mean and median score for all items in order to identify items with higher or lower than average impact and frequency and to assess response distributions. For the purposes of this descriptive analysis, we highlighted items with 50% or more of responses at the lowest or highest possible values as the cut-off value for which some items might be considered to show floor or ceiling characteristics, respectively. We also assessed missingness for each item and the time taken to complete the AIM. All descriptive analyses were also stratified by vertical/horizontal format.

#### Internal Consistency

To measure consistency of underlying concepts, Cronbach’s alpha (Cronbach 1951) was calculated for each of the domain scores. We specified a threshold of ≥ 0.7 (Nunnally and Bernstein 1994) to identify domains with a good internal consistency. Similarly, we also calculated Cronbach’s alpha for total AIM score and total scores based on just frequency items or impact items in order to assess whether AIM items contributing to a specific score measured the same construct. We calculated inter-domain correlations using Spearman’s correlation coefficient.

#### Convergent Validity

Pearson correlation coefficients were derived to assess the correlations between total SCQ and total AIM scores, as well as between the total SCQ and different domains of the AIM, and the domain scores of both. This approach was repeated for the RBS-R scores. For the SCQ, three domain scores were derived from item responses as per the SCQ scoring manual (Rutter et al. 2003). The domains are reciprocal social interaction, communication and repetition/stereotyped behavior. For RBS-R, factor analysis supports a total score, but also 2-, 3-, 4-, 5- and 6-factor solutions for domain-level scores, but this study utilized the 3-factor solution, which appears to have the most relevant conceptual structure (Mirenda et al. 2010): stereotypy restricted; self-injurious; compulsive, ritualistic, sameness.

We expected at least a moderate correlation (> 0.3) between the total SCQ/RBS-R and total AIM scores, as well as between the total SCQ/RBS-R and each of the AIM domain scores. Those domains for which we hypothesized the highest correlations (> 0.5) have been marked alongside the results for all domains in Table 3. Our hypotheses were based on domains which were conceptually related. Post-hoc, we recalculated correlations between SCQ and AIM communication domains within certain subgroups. The subgroups of interest were ASD individuals who were verbal or non-verbal only, as this limits the scoring range of the SCQ communication domain score, as well as those aged 4-5 years old, as this is the age range asked to focus on for half of the items of the SCQ: the other half have a lifetime perspective, e.g. ‘ever had’ (Rutter et al. 2003). In comparison, the RBS-R has no specified recall period and the AIM has a two-week recall period.

#### Factor Analysis

We summed frequency and impact scores for each of the 29 items which are needed to create the 5 domain scores proposed by the scale developers (Mazurek et al. 2018). We then fitted a 5-factor solution on those 29 items with Varimax rotation. Finally, we compared items with highest loadings on each factor in our solution, with the domains proposed. The purpose of our factor analysis was only to confirm the five domains suggested by the developers rather than to explore other potential factor solutions.

#### Known-Groups Analysis

Based on previous literature and clinical knowledge, pre-specified ‘known-groups’ were defined based on the following variables: (1) IQ score (Kanne et al. 2011; Mayes and Calhoun 2011); (2) proportion of school-time spent with typically developing peers (Rosen et al. 2019; Spaulding et al. Spaulding et al. 2017); (3) presence/absence of psychiatric comorbidity (Rosenberg et al. 2011); (4) received speech and language therapy (SLT) in the preceding 12 months (particularly relevant for communication domain); (5) caregiver reported overall health status of child (expected to be correlated with ASD severity if caregiver deems ASD symptoms relevant to overall health); (6) children who qualified for Medicaid despite family income greater than $75,000 per annum (to identify the subgroup who were Medicaid-eligible based on severity opposed to financial circumstance); (7) the number of non-drug therapies received for ASD in last 12 months (Rosen et al. 2019; Spaulding et al. 2017); (8) medication prescribed for ASD (assuming prescriptions are made for individuals with more severe symptoms, on average); (9) verbal/non-verbal ability (based on item 1 of the SCQ). More detailed definitions of these known-groups are provided in Table 1.

**Table 1:** Characteristics of Analysis Populations

	Completed AIM	Vertical electronic format	Horizontal electronic format	SCQ available	RBS-R available
N	4415	2933	1481	3064	3190
Child gender					
Male	3526 (79.9)	2341 (79.8)	1184 (79.9)	2426 (79.2)	2539 (79.6)
Female	864 (19.6)	569 (19.4)	295 (19.9)	625 (20.4)	637 (20.0)
Missing	25 (0.6)	23 (0.8)	2 (0.1)	13 (0.4)	14 (0.4)
Child age in years (mean (sd))	9.01 (3.90)	8.74 (3.92)	9.53 (3.80)	8.84 (3.88)	8.94 (3.87)
Child age in years					
3–4	618 (14.0)	474 (16.2)	144 (9.7)	451 (14.7)	439 (13.8)
5–9	1903 (43.1)	1281 (43.7)	622 (42.0)	1359 (44.4)	1417 (44.4)
10–14	1396 (31.6)	861 (29.4)	534 (36.1)	938 (30.6)	990 (31.0)
15–17	491 (11.1)	311 (10.6)	180 (12.2)	316 (10.3)	344 (10.8)
Missing	7 (0.2)	6 (0.2)	1 (0.1)	0 (0.0)	0 (0.0)
Caregiver age in years (mean (sd))	38.74 (7.20)	37.72 (6.90)	40.76 (7.31)	38.34 (7.11)	38.29 (7.07)
Caregiver relationship to child					
Mother	4091 (92.7)	2770 (94.4)	1321 (89.2)	2863 (93.4)	2985 (93.6)
Father	253 (5.7)	117 (4.0)	135 (9.1)	158 (5.2)	163 (5.1)
Legal guardian	42 (0.9)	24 (0.8)	18 (1.2)	25 (0.8)	24 (0.8)
Other	11 (0.2)	5 (0.2)	6 (0.4)	8 (0.3)	8 (0.3)
Unknown	18 (0.4)	17 (0.6)	1 (0.1)	10 (0.3)	10 (0.3)
US Region					
West	1116 (25.3)	739 (25.2)	376 (25.4)	782 (25.5)	811 (25.4)
Midwest	987 (22.4)	642 (21.9)	345 (23.3)	688 (22.5)	730 (22.9)
Northeast	680 (15.4)	448 (15.3)	232 (15.7)	459 (15.0)	490 (15.4)
South	1624 (36.8)	1100 (37.5)	524 (35.4)	1128 (36.8)	1153 (36.1)
Unknown	8 (0.2)	4 (0.1)	4 (0.3)	7 (0.2)	6 (0.2)
IQ					
IQ score 70 or below	390 (8.8)	251 (8.6)	139 (9.4)	250 (8.2)	262 (8.2)
IQ score between 71 and 99	489 (11.1)	300 (10.2)	189 (12.8)	345 (11.3)	348 (10.9)
IQ score 100 or above	670 (15.2)	424 (14.5)	246 (16.6)	466 (15.2)	489 (15.3)
Don’t know or Never done	2866 (64.9)	1958 (66.8)	907 (61.2)	2003 (65.4)	2091 (65.5)
School time spent with TD peers					
Full time special education	940 (21.3)	634 (21.6)	306 (20.7)	623 (20.3)	650 (20.4)
Less than 30%	829 (18.8)	555 (18.9)	274 (18.5)	580 (18.9)	603 (18.9)
More than 30%, less than 60%	510 (11.6)	341 (11.6)	169 (11.4)	357 (11.7)	372 (11.7)
More than 60%	1997 (45.2)	1304 (44.5)	693 (46.8)	1401 (45.7)	1465 (45.9)
Unknown	139 (3.1)	99 (3.4)	39 (2.6)	103 (3.4)	100 (3.1)
Other psychiatric comorbidity					
Yes	2076 (47.0)	1381 (47.1)	694 (46.9)	1428 (46.6)	1485 (46.6)
No	2235 (50.6)	1480 (50.5)	755 (51.0)	1560 (50.9)	1633 (51.2)
Don’t know/missing	104 (2.4)	72 (2.5)	32 (2.2)	76 (2.4)	72 (2.3)
SLT received in last 12 months					
Yes	3177 (72.0)	2126 (72.5)	1051 (71.0)	2192 (71.5)	2284 (71.6)
No	1238 (28.0)	807 (27.5)	430 (29.0)	872 (28.5)	906 (28.4)
Overall child health					
Excellent, very good or good	4259 (96.5)	2818 (96.1)	1440 (97.2)	2952 (96.3)	3092 (96.9)
Fair or poor	150 (3.4)	109 (3.7)	41 (2.8)	112 (3.7)	97 (3.0)
Missing	6 (0.1)	6 (0.2)	0 (0.0)	0 (0.0)	1 (0.0)
High income and Medicaid coverage^a^					
Yes	415 (9.4)	248 (8.5)	167 (11.3)	262 (8.6)	279 (8.7)
No	3793 (85.9)	2568 (87.6)	1225 (82.7)	2675 (87.3)	2776 (87.0)
Unknown	207 (4.7)	117 (4.0)	89 (6.0)	127 (4.1)	135 (4.2)
Non-drug ASD therapies in last 12 months					
4 or fewer	3700 (83.8)	2460 (83.9)	1239 (83.7)	2581 (84.2)	2686 (84.2)
5 or more	715 (16.2)	473 (16.1)	242 (16.3)	483 (15.8)	504 (15.8)
Prescription drug for ASD					
Yes	1453 (32.9)	966 (32.9)	487 (32.9)	986 (32.2)	1041 (32.6)
No	2920 (66.1)	1938 (66.1)	981 (66.2)	2052 (67.0)	2118 (66.4)
Don’t know	42 (1.0)	29 (1.0)	13 (0.9)	26 (0.8)	31 (1.0)
Verbal^b^					
Yes	2559 (58.0)	1699 (57.9)	859 (58.0)	2559 (83.5)	2155 (67.6)
No	505 (11.4)	378 (12.9)	127 (8.6)	505 (16.5)	416 (13.0)
Unknown	1351 (30.6)	856 (29.2)	495 (33.4)	0 (0.0)	619 (19.4)

Numbers indicate n(%) unless specified. One respondent had an unidentified screen size. Overall child health was caregiver reported

Vertical format: e.g. on mobile devices; Horizontal format: e.g. desktop computers and laptops

*SLT* speech and language therapy, *TD* typically developing

^a^’Yes’ defined as > $75,000 household income per year but still qualified for Medicaid

^b^As defined by question 1 of Social Communication Questionnaire (SCQ)

n = 2571 participants completed all of AIM, SCQ and RBS-R

We summarized mean and median scores within each level of each known-group and conducted analysis of variance (ANOVA) to see if those differences were statistically significant (p < 0.05). We produced both crude and age-adjusted ANOVA results based on the total AIM scores, total frequency/impact scores and individual domain scores.

The range of possible scores for the total AIM was 82–410. For both frequency and impact domains the possible range was 41–205. For each of the domains, the possible ranges were: 16–80 for repetitive behavior; 12–60 for communication; 12–60 for atypical behavior; 10–50 for social reciprocity; 8–40 for peer interaction.

#### Clinically Important Responder (CIR) Estimates

As data were collected cross-sectionally we estimated clinically important responder (CIR) thresholds (see Coon and Cappelleri 2016) for the total AIM scores and domain scores using distribution-based methods. Specifically the estimates were based on one-fifth and one-half of standard deviations (Fayers and Hays 2014; Norman et al. 2003). Prior to generating estimates, we rescaled the maximum range of total and domains scores to 0–100 points. This was done in order to make the magnitude of CIR estimates easier to compare across domains. For completeness we also presented CIR estimates based on raw scores, and we repeated the analysis by age and IQ strata to check for homoscedasticity.

## Results

### Cohorts and Descriptive Analysis

**Fig. 1:**
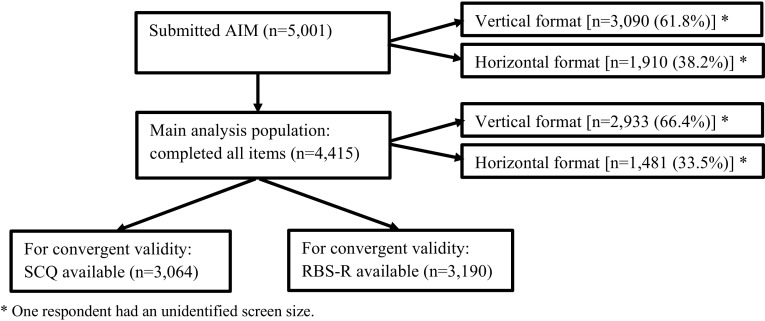
Flow Chart of Analysis Populations

Figure 1 and Table 1 display the flow chart of key populations and their characteristics, respectively. The majority of participants completed all items (n = 4415; 88.3%). This ‘completers’ group was used as the main analysis group. Around two-thirds of completers (66.4%) took part in the AIM in vertical layout.

Respondents with complete AIM were mainly mothers (92.7%) with a mean (SD) age 38.74 (7.20) years. All 50 states of the US were represented as well as some overseas territories. Children with ASD had a mean (SD) age of 9.01 (3.90) and were mainly male (79.9%). Almost a quarter of children (23.1%) attended full time special education school, while 45.2% spent between 60%-100% of school time with typically developing peers. Of those with SCQ available, 83.5% were verbal (according to item 1 of the SCQ). The only qualitatively notable difference between caregivers who used the vertical instead of horizontal format was their slightly younger mean age (37.7 vs. 40.8 years). Furthermore, there were no notable differences for completers, non-completers, and those which had SCQ and/or RBS-R data available for linkage.

The median time to complete the AIM was 7.08 min [IQR 5.53–9.82]. The mean time was just over one minute faster for completers on the horizontal format (median [IQR] 6.28 min [4.90–8.63]) versus the vertical format (median [IQR] 7.47 min [5.97–10.45]). A minority (4.1% in both vertical and horizontal format) took over one hour to complete all questions.

### Item Level Analysis

Full item level analyses are summarized in supplementary Table S1. Responses to most items were approximately normally distributed. None of the items had a ceiling effect, but 5 had a floor effect which was defined by a median response of 1. Namely these items were: Q3 “lined things up” [impact only, repetitive behavior domain]: Q5 “used hand over hand” [frequency and impact; communication domain]: Q27 “used made-up or private language” [frequency and impact; communication domain].

Disregarding missing values, the item with highest (most severe) mean score (3.90) was Q38 “engaged in chit-chat [frequency; social reciprocity domain]. Furthermore, the top five highest scoring items were all frequency questions and only three of the top 20 highest scoring (mean ≥ 3.02) were impact questions. Only two of the 20 lowest scoring items (mean ≤ 2.28) were frequency related. Mean scores for each item were not systematically higher or lower based on the vertical or horizontal layout.

Overall, there was very little missing data on an item-by-item basis. Some questions had as little as 10 missed responses from the whole sample (0.20%). Q36 “showed interest in others” [impact] was most frequently missed but still only for 76 participants (1.52%). All items were more often missing on the horizontal format, however with 2.46% being the highest rate of missing data in this layout (Q36 impact). In general, impact questions were more commonly missing than frequency questions.

### Internal Consistency

Cronbach alpha for the total AIM score was 0.96, which is well above the threshold of 0.7, which we pre-specified would identify scores with a good internal consistency. Frequency items and impact items also showed high internal consistency (0.96 and 0.95 respectively), as did each of the individual domains (from 0.79 for social reciprocity to 0.91 for communication). The median (IQR) of all inter-item correlations was r = 0.15 (0.22–0.30) and only the correlation between frequency and impact scores for Q6 “problems with speech” was higher than 0.90. These results indicate little item redundancy.

All domains were positively and moderately inter-related according to Spearman’s rank coefficient (Table 2). The weakest relationship was between Repetitive Behavior and Social Reciprocity (0.39). The strongest relationship was between Repetitive Behavior and Atypical Behavior (0.67). Domain correlations were very similar with both Spearman and Pearson correlation methods, indicating that relationships between domain scores were linear.

**Table 2:** AIM Inter-domain Spearman–rank correlations

	Repetitive behavior	Communication	Atypical behavior	Social reciprocity	Peer interaction
Repetitive behavior	–	0.52	0.67	0.39	0.43
Communication		–	0.45	0.54	0.48
Atypical behavior			–	0.51	0.58
Social reciprocity				–	0.63

### Convergent Validity

The total AIM score showed good convergent validity with the total SCQ score (r = 0.55, Table 3). Each individual AIM domain was also positively correlated (r ≥ 0.34) with the total SCQ score. As hypothesized, the SCQ Reciprocal Social Interaction domain has highest correlations with the AIM Social Reciprocity (0.48) and Peer Interaction (0.45) domains. Also as expected, the SCQ Repetition/Stereotyped Behavior domain had the strongest relationship with AIM domains of Repetitive Behavior (0.48) and Atypical Behavior (0.34). None of the SCQ-AIM domain-domain relationships met the threshold of 0.5 however, and specifically against our expectations, the SCQ Communication domain was least correlated with the AIM Communication domain (0.18). In sensitivity analyses this correlation was raised to 0.34 in verbal children and 0.25 in non-verbal children. When restricting to a 4 to 5 years old age-range, the correlation was 0.19.

**Table 3:** Convergent Validity (Pearson’s correlations) between AIM Domains and SCQ and RBS-R Domains

AIM	SCQ (n = 3064)				RBS-R (n = 3190)			
	Reciprocal social interaction	Communication	Repetition/Stereotyped Behavior	Total	Stereotypy restricted	Self-injurious	Compulsive, ritualistic, Sameness	Total
AIM domain-repetitive behavior	0.34	0.25	0.48^a^	0.46	0.74^a^	0.46	0.66^a^	0.74
AIM domain-communication	0.37	0.18^ab^	0.15	0.34	0.44	0.29	0.23	0.33
AIM domain-atypical behavior	0.38	0.29	0.34^a^	0.45	0.51^a^	0.42	0.55^a^	0.59
AIM domain-social reciprocity	0.48^a^	0.33	0.16	0.45	0.31	0.24	0.25	0.30
AIM domain-peer interaction	0.45^a^	0.28	0.17	0.41	0.31	0.26	0.26	0.31
AIM frequency				0.60				0.63
AIM impact				0.45				0.58
AIM total				0.55				0.64

Exact date of SCQ/RBS-R unknown so analysis population restricted to where the child’s age (in years) at time of SCQ/RBS-R is within 1 year of age at time of AIM

Total SCQ and total RBS-R were expected to have correlations > 0.3 with all AIM domains and summary scores

^a^Domain correlations with pre-specified expected highest correlations of > 0.5

^b^Result was 0.34 in verbal group, 0.25 in non-verbal group and 0.19 in age group 4-5 years

The RBS-R total score had a strong positive correlation with the total AIM score (0.64). It also had good correlation (≥ 0.30) with all AIM domains, frequency and impact scores. Furthermore, for the RBS-R and AIM, all domain-domain correlations were positive, and were strongest (between 0.51 and 0.74) in the 4 pre-hypothesized cases. Results for both SCQ and RBS-R remained stable when restricting the analysis population to those children who were exactly the same age (in years) at the time of SCQ/RBS-R and AIM (opposed to within 1-year, as per main analyses; see Table S3).

### Factor Analysis

Table 4 provides a detailed comparison of the proposed factors (Mazurek et al. 2018) and factors found in our confirmatory analysis. The Communication domain was replicated perfectly in our data. The proposed 6 items for this domain all loaded highest on the third factor produced by our data and no other item loaded highest on this same factor. Other well pronounced and well reproduced latent concepts were Repetitive Behavior and Social Reciprocity. All items proposed for these domains loaded highest on factor 1 and factor 2 in our data, respectively. The only additional item with highest loading on factor 2 was Q32 “had positive response to approach”, which was supposed to be part of the Peer Interaction domain. Q32 also had a high loading on factor 4 however, and factor 4 otherwise only had highest loadings of the other 3 of the 4 items representing the Peer Interaction domain. Hence Peer Interaction was also well reproduced as a latent variable. Finally, 3 of the 6 items expected to load together to form the Atypical Behavior domain indeed did load together in a distinct fifth factor. The other 3 items however loaded highest on factor 1, showing some similarity with the Repetitive Behavior concept. The first 3 factors collectively explained 37.1% of total variance in the data. Five factors explained 48.4%.

**Table 4:** Factor Analysis and Specified Domains of the AIM

Proposed Domain^a^	Item	Basic item content	Factor 1	Factor 2	Factor 3	Factor 4	Factor 5
		Proportion of variance explained	**(14.4%)**	**(11.5%)**	**(11.2%)**	**(6.4%)**	**(4.8%)**
Repetitive behavior	10	Repeated actions	**0.59**	0.11	0.37	0.16	0.02
	14	Problems with repetitive behaviors	**0.56**	0.15	0.17	0.20	0.27
	1	Fascination with parts	**0.58**	0.11	0.25	0.12	-0.01
	13	Attached to objects	**0.58**	0.06	0.15	0.12	0.08
	7	Engaged in rituals or routines	**0.67**	0.14	0.05	0.00	0.16
	12	Exhibited repetitive hand and finger movements	**0.46**	0.09	0.28	0.14	0.03
	15	Avoided sounds, textures, or smells	**0.55**	0.12	0.07	0.08	0.21
	3	Lined things up	**0.52**	0.06	0.13	-0.04	-0.06
Social reciprocity	39	Exhibited range of facial expressions	0.15	**0.68**	0.13	0.08	0.08
	34	Used gestures to communicate	0.13	**0.59**	0.24	0.17	0.05
	30	Shared enjoyment	0.13	**0.59**	0.22	0.20	0.08
	38	Engaged in chit–chat	0.04	**0.59**	0.38	0.24	0.09
	41	Made eye contact	0.22	**0.51**	0.02	0.05	0.18
Communication	6	Problems with speech	0.14	0.15	**0.82**	0.06	0.12
	18	Problems with communication	0.17	0.24	**0.68**	0.13	0.40
	23	Had difficulty with pronouns	0.20	0.18	**0.74**	0.06	0.00
	5	Used hand over hand	0.29	0.16	**0.53**	0.14	-0.08
	29	Engaged in reciprocal communication	0.07	0.51	**0.56**	0.21	0.09
	27	Used a made-up or private language?	0.34	0.09	**0.38**	0.07	0.01
Peer interaction	36	Showed interest in others	0.12	0.52	0.16	**0.59**	0.05
	32	Had positive response to approach	0.18	**0.50**	0.11	0.43	0.18
	28	Played with same aged peers	0.09	0.38	0.15	**0.64**	0.15
	9	Was withdrawn from others	0.26	0.25	0.20	**0.59**	0.32
Atypical behavior	26	Problems in social interactions	0.28	0.28	0.17	0.36	**0.53**
	22	Resistant to changes	**0.61**	0.19	-0.04	0.02	0.29
	21	Had difficulty with affection	**0.37**	0.33	-0.02	0.10	0.26
	16	Was aloof	0.34	0.30	0.12	0.22	**0.41**
	8	Had odd vocal tone or pitch	**0.44**	0.12	0.23	0.10	0.24
	4	Demonstrated odd responses	0.42	0.15	0.04	0.18	**0.44**

All other rows represent the loadings of each item on each factor. The largest loading per item is highlighted in bold font

^a^Mazurek et al. 2018. The first row (with numbers in brackets) gives the percentage of total variance in the dataset, which is explained by each factor

### Known-Group Analysis

For the patients who completed all items, the mean (median) total AIM score was 220.8 (219). In general, frequency items received higher scores than impact items [119.9 (120) vs.100.9 (99)]. Mean and median scores for the five domains were; Repetitive Behavior: 41.3 (40); Communication: 30.7 (28); Atypical Behavior: 34.8 (35); Social Reciprocity 27.1 (27); Peer Interaction 22.9 (23). All of the above summary scores were approximately normally distributed.

Mean scores for total AIM, frequency, impact and all domains increased monotonically from high IQ to low IQ. These associations of low IQ and greater ASD severity were statistically significant in ANOVA analysis (p < 0.01 in all domains). AIM scores were similar between those in full time special education and those who spent less than 30% of school-time with typically developing peers. Otherwise, AIM scores increased with higher proportion of special-education activity and all differences were statistically significant (p < 0.01).

**Fig. 2:**
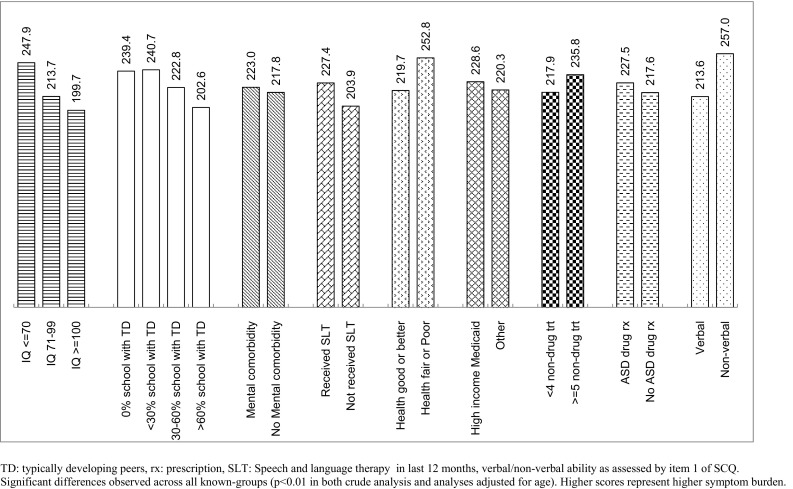
Mean Total AIM Score by Known-groups

**Fig. 3:**
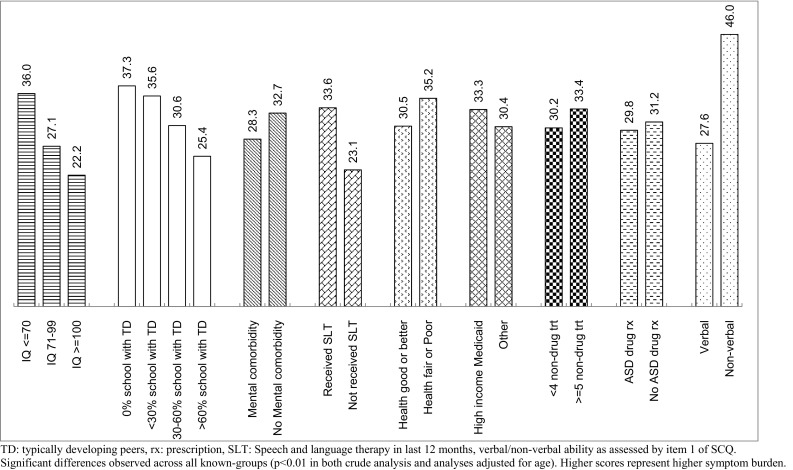
Mean AIM Communication Domain Score by Known-groups

Other ‘known-groups’ were binary-categorized. Both total AIM score (Fig. 2) and impact score (supplementary Figure S2) were able to differentiate between all pre-defined known-groups (p < 0.01). All such associations were directionally as expected, with higher scores in the group expected to have more severe ASD. The largest difference in mean total AIM score was between verbal and non-verbal children (257 vs 214, respectively). The frequency score also differentiated between all known groups (p < 0.01) except for those children with or without another psychiatric comorbidity (p = 0.41, Figure S1). Mean scores for the Communication (Fig. 3) and Peer Interaction (Figure S6) domains were significantly different (p < 0.01) between levels of all 9 pre-defined known-groups. Repetitive Behavior, Social Reciprocity and Atypical Behavior domains significantly (p < 0.01) distinguished between levels of 8, 8 and 7 of the 9 known-groups respectively, too (see supplementary Figures S3-S5). None of the results for known-groups were altered by adjusting for age, i.e. p-values always remained stable (either ≥ 0.05, between 0.01 and 0.05, or < 0.01). Results for a total AIM score based on only 29 items were very similar to those based on all 41 items.

### Clinically Important Response Estimates

For the total AIM score, the CIR estimate ranged from 3.30% to 8.25% (Table 5). This corresponded to a change of between 10.8 and 27.1 points on the raw scale (Table S2). The CIR estimate range for the frequency score was between 3.21% and 8.04% and between 3.74% and 9.34% for the impact score. Of the domains, Social Reciprocity had the least variability and hence the smallest estimates for the CIR (3.67% to 9.16%). All other domains had CIR estimates ranging between 4.20% and 4.96% at the lower end, and between 10.49% and 12.41% at the upper end.

**Table 5:** Estimates for Clinically Important Responses of the AIM scores, overall and by age and IQ group (rescaled scores 0-100)

AIM	n	Total	Frequency	Impact	Repetitive behavior	Communi-cation	Atypical behavior	Social reciprocity	Peer interaction
Overall	4415	3.30–8.25	3.21–8.04	3.74–9.34	4.29–10.73	4.96–12.41	4.20–10.49	3.67–9.16	4.45–11.12
3–4 years	618	3.04–7.60	2.91–7.26	3.59–8.98	4.05–10.12	4.35–10.87	4.08–10.21	3.64–9.09	4.51–11.27
5–9 years	1903	3.33–8.33	3.23–8.08	3.77–9.43	4.24–10.61	4.87–12.16	4.16–10.40	3.76–9.40	4.51–11.27
10–14 years	1396	3.25–8.12	3.21–8.02	3.62–9.06	4.29–10.72	4.51–11.26	4.16–10.39	3.55–8.87	4.33–10.81
15–17 years	491	3.42–8.55	3.27–8.16	3.91–9.79	4.41–11.02	4.53–11.33	4.47–11.17	3.57–8.93	4.41–11.02
IQ < 70	390	3.30 - 8.25	3.10–7.76	3.91–9.78	4.40–11.01	4.67–11.68	4.41–11.03	3.41–8.52	4.38–10.94
IQ 71–99	489	2.88–7.19	2.80–7.00	3.30–8.25	4.03–10.07	3.73–9.32	3.76–9.39	3.39–8.47	4.13–10.32
IQ > 100	670	2.92–7.29	2.88–7.19	3.30–8.26	4.09–10.22	3.11–7.78	4.00–10.01	3.29–8.21	4.15–10.38

Estimates for CIR are 0.2–0.5 times standard deviation. Prior to calculation of CIR, scores were rescaled to represent percentage change across the full range of possible scores. See methods section for details. See supplementary Table S2 for corresponding raw scores changes. Participants with missing age or IQ data were excluded from respective analyses

The largest change in variability across strata was for the Communication domain and IQ level. CIR estimates decreased monotonically from low to high IQ (11.69% for IQ < 70, 7.78% for IQ > 100; upper estimates). This corresponded to a 3.7 to 5.6-point difference on the raw scale (in which a maximum change of 48 points is possible). This example aside, the data had stable variance across IQ and age ranges, because estimates of variability were generally only slightly higher in the groups with smallest sample size (IQ < 70 and age 15–17 years). Generally, variance was slightly smaller within children of similar IQ, rather than of similar age.

## Discussion

To our knowledge, this study represents the largest fielding of the AIM to an ASD population to date. Our main findings were the estimates of thresholds of clinical importance and the ability of the AIM to separate known groups of children with ASD. We also believe our study represents the first investigations of these concepts for the AIM. Overall, the CIR for the total AIM score was estimated to be in the range of 3.30–8.25%, corresponding to 10.8–27.1 points of the full 82 to 410 score range. Total AIM scores and most domain scores were generally homogeneous across age and IQ strata, with the only marked exception being that there was more variation in communicative abilities of children with low IQ. Of 9 pre-defined known-groups, the AIM total score statistically differentiated all of them. Mean scores on each of the domains separated almost all known-groups too. Moreover, according to the lower bound for CIR estimates from above, the majority of these differences represented clinical meaningfulness. Even for the Communication domain, which had the largest CIR estimates relative to scale, the lower estimate (5.0%) was surpassed in all but one of the known-groups (yes/no to current prescription drug for ASD). The more stringent upper estimate of 12.4% was even achieved in 4 of the 9 known-groups. Namely these groups were: school time with typically developing peers, IQ strata, verbal ability and participation in SLT. In all, these results do provide some confidence that the AIM should be able to respond to symptom changes over time. However, it is uncertain whether any intervention (pharmacological or non-pharmacological) could change such fundamental personal characteristics as represented by our known-groups. Likely our lower estimates for CIR are a most reasonable goal. A limitation of the CIR results is that only distribution-based estimates were generated due to a lack of follow-up data and an appropriate anchor, such as caregiver reported assessment of change. Therefore, further evaluation is required to test empirically the estimates generated.

Our sample, on the whole, was very similar to those used in previous AIM studies (Kanne et al. 2014; Mazurek et al. 2018), in that respondents were mainly mothers of the child with ASD (around 90%), and families lived at various locations across the US. Children with ASD in each study were mainly male (between 80%-84%) and of similar age (between 2-14, 2-16, or 3-17 years). A key difference however, was that we fielded the AIM electronically, rather than on paper. We used this opportunity for secondary objectives of retesting other psychometric properties of the AIM in this format.

Importantly, there were no striking differences in the characteristics of participants or their responses, based on if they used the vertical or horizontal version of the questionnaire. Our data in the most part also confirmed the suitability of an underlying 5-factor structure of the AIM proposed by Mazurek et al. (2018). Items proposed for the Repetitive Behavior, Communication, and Social Reciprocity domains all loaded highly and separately from each other. These first 3 domains accounted for almost 40% of the variability in our data: impressive, given the heterogeneity of symptoms on the autism spectrum. In addition, the magnitude of variability explained by each of these domains was almost equal (11-15% each) and in line with the three core symptoms of ASD.

External convergent validity of the AIM total scores and most domain scores was also demonstrated. Specifically, both Repetitive Behavior and Atypical Behavior correlated highly (r > 0.50) with the RBS-R domains of similar concepts. This is despite the RBS-R having no specific recall period, but the AIM having a two-week recall. Correspondence of the AIM to the SCQ total score was also high. Four out of 5 domain-domain relationships that were expected to generate the highest correlation coefficients did exactly that, albeit not to the extent hypothesized (r = 0.34 to 0.48). Only the relationship between AIM Communication and SCQ Communication domains were at odds to the expected. The correlation was still positive but of modest magnitude (r = 0.18). Sensitivity analysis in children aged 4-5 years – which is the age range asked to focus on for some items of the SCQ (Rutter et al. 2003) - did not improve this (r = 0.19). Nonetheless, the AIM Communication domain does represent a clear latent variable, given the perfect representation of this domain mentioned in factor-analysis results above. One explanation is that the AIM and SCQ Communication domains measure subtly different concepts. AIM Communication items mainly already assume verbal ability with some questions relating to concepts like made-up languages, use of pronouns, and reciprocal communication. In contrast some SCQ items relating to communication are specifically omitted for non-verbal children (Rutter et al. 2003). An alternative explanation is that the AIM directs caregivers to recall symptom severity over the last two weeks, whereas SCQ items have a lifetime perspective. A limitation of this study is that the SCQ and RBS-R surveys were not taken at the same time as the AIM, hence it is difficult to evaluate if non-concordance is due to differences in conceptual constructs or is due to actual differences in symptom severity at time of survey completion. Another more general limitation of the study is that all data are caregiver-reported and therefore some demographic and personal characteristics (e.g. IQ score) may be based on estimates only.

### Future Research and Use of the AIM

Our CIR estimates above can be used to inform studies wishing to use the AIM in the near future. Better still would be to have repeated follow up in the same patients in order to also estimate CIR based on anchor based approaches (Engel et al. 2018; Wright et al. 2012). This is a possibility, as all data from this current study will be made available via SPARK.

Missing data was slightly more common in the horizontal layout and for impact questions but otherwise was seldom and unsystematic. The most commonly skipped item was only done so by 1.52% of respondents, but overall we had to exclude around 10% of the sample, as the developers currently offer no advice on dealing with missing data (Kanne et al. 2014; Mazurek et al. 2018). Given our findings that missing item level data is infrequent, that the AIM has good internal consistency (a = 0.96), and that most items are normally distributed, we recommend imputing missing items by multiple imputation (perhaps only excluding some observations with missing data above a pre-defined threshold value of e.g. 20%). This approach has worked well for the AIM elsewhere (Monz et al. 2019).

Throughout our analysis, the Frequency and Impact summary scores also displayed good psychometric properties. This means that in addition to total and domain scores, future researchers could use the summed Frequency or Impact scores, depending on their specific question. In particular, the Frequency score might be more useful, because Impact may be more easily affected by other things than interventions, such as coping mechanisms built into everyday life. Furthermore, if an items frequency score is low, then the impact question may become redundant.

Electronically reported outcome measures have added benefits over paper-based measures. These include the avoidance of data entry errors, increased willingness of respondents to share sensitive information, and quicker access to this data for research (Deshpande et al. 2011). Electronic measures can also be completed remotely. A clear advantage of the AIM, is the limited time needed to complete it (median time: 7 min). This coupled with high overall participation rate in our study (Monz et al. 2019) demonstrates that caregivers are comfortable completing the AIM in such a way. This means that the AIM could potentially enable cheaper and low burden monitoring of severity changes as well as effectiveness of interventions in a real-world setting.

## Conclusion

Our study provides estimates of thresholds of clinical importance for the AIM, as well as some indication that the AIM can distinguish between known groups of children with ASD. Our results also confirm the validity of the AIM based on other important psychometric properties. When administered electronically, the AIM offers a quick and relatively inexpensive method for caregivers to report core symptoms of children with ASD, including communication deficits, difficulties with social interactions and repetitive behaviors.

## Electronic supplementary material

Below is the link to the electronic supplementary material.

Supplementary material 1 (DOCX 50 kb)
