# Correction to: Psychometric Validation of the Autism Impact Measure (AIM)

**DOI:** 10.1007/s10803-019-04076-z

**Published:** 2019-05-28

**Authors:** Richard Houghton, Brigitta Monz, Kiely Law, Georg Loss, Stephanie Le Scouiller, Frank de Vries, Tom Willgoss

**Affiliations:** 1 Personalized Health Care Data Science, Real World Data, F. Hoffmann-La Roche Ltd, Grenzacherstrasse. 124, 4070 Basel, Switzerland; 2 School CAPHRI, Maastricht University, Maastricht, The Netherlands; 3 Kennedy Krieger Institute, 707 North Broadway, Baltimore, MD 21205 USA; 4 Johns Hopkins University School of Medicine, 733 North Broadway, Baltimore, MD 21205 USA; 5 Patient Centered Outcomes Research, Biometrics, Roche Products, Ltd, Falcon Way, Welwyn Garden City, UK; 6 Department of Clinical Pharmacy & Toxicology, Maastricht UMC+, Maastricht, The Netherlands

## Correction to: Journal of Autism and Developmental Disorders 10.1007/s10803-019-04011-2

The article Psychometric Validation of the Autism Impact Measure (AIM), written by Richard Houghton, Brigitta Monz, Kiely Law, Georg Loss, Stephanie Le Scouiller, Frank de Vries and Tom Willgoss was originally published electronically on the publisher’s internet portal (currently SpringerLink) on 09 April 2019 without open access.With the author(s)’ decision to opt for Open Choice the copyright of the article changed on May 2019 to © The Author(s) 2019 and the article is forthwith distributed under the terms of the Creative Commons Attribution 4.0 International License (http://creativecommons.org/licenses/by/4.0/), which permits use, duplication, adaptation, distribution and reproduction in any medium or format, as long as you give appropriate credit to the original author(s) and the source, provide a link to the Creative Commons license and indicate if changes were made.

