# Supplementary material for: Psychometric Validation of the Autism Impact Measure (AIM)
Source: J Autism Dev Disord. 2019 Apr 9;49(6):2559–70. doi: 10.1007/s10803-019-04011-2 (PMC6546866; doi:10.1007/s10803-019-04011-2)
Supplement: Supplementary file 1 — Supplementary material 1 (DOCX 50 kb) [file 10803_2019_4011_MOESM1_ESM.docx]

Supplementary tables and figures

Table S1: Item scores (descending order based on mean)

| Item | Basic Content | Component | Mean | Mean Vertical format | Mean Horizontal format | Mean Difference between formats |
| --- | --- | --- | --- | --- | --- | --- |
| 38 | Engaged in chit–chat | Frequency | 3.90 | 3.91 | 3.87 | 0.04 |
| 26 | Problems in social interactions | Frequency | 3.61 | 3.59 | 3.63 | -0.04 |
| 35 | Comforted others | Frequency | 3.61 | 3.66 | 3.50 | 0.16 |
| 20 | Circumscribed interest | Frequency | 3.46 | 3.50 | 3.39 | 0.11 |
| 18 | Problems with communication | Frequency | 3.44 | 3.44 | 3.42 | 0.02 |
| 26 | Problems in social interactions | Impact | 3.40 | 3.38 | 3.42 | -0.04 |
| 31 | Played cooperatively | Frequency | 3.39 | 3.39 | 3.40 | -0.01 |
| 4 | Demonstrated odd responses | Frequency | 3.35 | 3.35 | 3.35 | 0.00 |
| 18 | Problems with communication | Impact | 3.34 | 3.36 | 3.31 | 0.05 |
| 14 | Problems with repetitive behaviors | Frequency | 3.32 | 3.36 | 3.26 | 0.10 |
| 7 | Engaged in rituals or routines | Frequency | 3.25 | 3.32 | 3.11 | 0.21 |
| 28 | Played with same aged peers | Frequency | 3.24 | 3.23 | 3.25 | -0.02 |
| 29 | Engaged in reciprocal communication | Frequency | 3.16 | 3.16 | 3.16 | 0.00 |
| 8 | Had odd vocal tone or pitch | Frequency | 3.10 | 3.12 | 3.04 | 0.08 |
| 22 | Resistant to changes | Frequency | 3.08 | 3.09 | 3.06 | 0.03 |
| 33 | Exhibited social smile | Frequency | 3.08 | 3.08 | 3.07 | 0.01 |
| 4 | Demonstrated odd responses | Impact | 3.07 | 3.07 | 3.06 | 0.01 |
| 9 | Was withdrawn from others | Frequency | 3.07 | 3.07 | 3.06 | 0.01 |
| 32 | Had positive response to approach | Frequency | 3.05 | 3.06 | 3.03 | 0.03 |
| 6 | Problems with speech | Frequency | 3.02 | 3.06 | 2.95 | 0.11 |
| 40 | Shared interest | Frequency | 2.98 | 3.02 | 2.89 | 0.13 |
| 36 | Showed interest in others | Frequency | 2.97 | 2.98 | 2.94 | 0.04 |
| 14 | Problems with repetitive behaviors | Impact | 2.96 | 2.98 | 2.91 | 0.07 |
| 39 | Exhibited range of facial expressions | Frequency | 2.94 | 2.94 | 2.93 | 0.01 |
| 22 | Resistant to changes | Impact | 2.92 | 2.94 | 2.88 | 0.06 |
| 29 | Engaged in reciprocal communication | Impact | 2.92 | 2.95 | 2.84 | 0.11 |
| 37 | Used imagination | Frequency | 2.92 | 2.94 | 2.88 | 0.06 |
| 15 | Avoided sounds, textures, or smells | Frequency | 2.91 | 2.96 | 2.80 | 0.16 |
| 2 | Fascinated with looking, feeling, smelling, licking | Frequency | 2.90 | 2.92 | 2.86 | 0.06 |
| 6 | Problems with speech | Impact | 2.90 | 2.95 | 2.82 | 0.13 |
| 20 | Circumscribed interest | Impact | 2.88 | 2.92 | 2.81 | 0.11 |
| 16 | Was aloof | Frequency | 2.87 | 2.87 | 2.87 | 0.00 |
| 7 | Engaged in rituals or routines | Impact | 2.86 | 2.91 | 2.75 | 0.16 |
| 31 | Played cooperatively | Impact | 2.86 | 2.87 | 2.84 | 0.03 |
| 41 | Made eye contact | Frequency | 2.86 | 2.88 | 2.84 | 0.04 |
| 9 | Was withdrawn from others | Impact | 2.84 | 2.85 | 2.82 | 0.03 |
| 13 | Attached to objects | Frequency | 2.80 | 2.85 | 2.69 | 0.16 |
| 34 | Used gestures to communicate | Frequency | 2.80 | 2.81 | 2.79 | 0.02 |
| 12 | Exhibited repetitive hand and finger movements | Frequency | 2.77 | 2.79 | 2.71 | 0.08 |
| 32 | Had positive response to approach | Impact | 2.77 | 2.79 | 2.74 | 0.05 |
| 11 | Collected things | Frequency | 2.75 | 2.78 | 2.69 | 0.09 |
| 19 | Had odd approaches | Frequency | 2.75 | 2.74 | 2.76 | -0.02 |
| 1 | Fascination with parts | Frequency | 2.74 | 2.80 | 2.63 | 0.17 |
| 24 | Demonstrated repetitive phrases | Frequency | 2.74 | 2.76 | 2.69 | 0.07 |
| 23 | Had difficulty with pronouns | Frequency | 2.73 | 2.79 | 2.60 | 0.19 |
| 16 | Was aloof | Impact | 2.70 | 2.71 | 2.66 | 0.05 |
| 25 | Exhibited echoed speech | Frequency | 2.69 | 2.73 | 2.60 | 0.13 |
| 38 | Engaged in chit–chat | Impact | 2.64 | 2.61 | 2.68 | -0.07 |
| 15 | Avoided sounds, textures, or smells | Impact | 2.60 | 2.66 | 2.48 | 0.18 |
| 19 | Had odd approaches | Impact | 2.60 | 2.58 | 2.63 | -0.05 |
| 41 | Made eye contact | Impact | 2.58 | 2.56 | 2.56 | 0.02 |
| 8 | Had odd vocal tone or pitch | Impact | 2.55 | 2.55 | 2.54 | 0.01 |
| 30 | Shared enjoyment | Frequency | 2.55 | 2.56 | 2.52 | 0.04 |
| 10 | Repeated actions | Frequency | 2.54 | 2.60 | 2.43 | 0.17 |
| 36 | Showed interest in others | Impact | 2.51 | 2.50 | 2.54 | -0.04 |
| 17 | Exhibited repetitive whole body movements | Frequency | 2.50 | 2.50 | 2.49 | 0.01 |
| 28 | Played with same aged peers | Impact | 2.43 | 2.40 | 2.49 | -0.09 |
| 23 | Had difficulty with pronouns | Impact | 2.36 | 2.41 | 2.25 | 0.16 |
| 24 | Demonstrated repetitive phrases | Impact | 2.34 | 2.36 | 2.30 | 0.06 |
| 33 | Exhibited social smile | Impact | 2.33 | 2.30 | 2.38 | -0.08 |
| 3 | Lined things up | Frequency | 2.32 | 2.36 | 2.24 | 0.12 |
| 30 | Shared enjoyment | Impact | 2.31 | 2.30 | 2.32 | -0.02 |
| 2 | Fascinated with looking, feeling, smelling, licking | Impact | 2.28 | 2.31 | 2.22 | 0.09 |
| 39 | Exhibited range of facial expressions | Impact | 2.27 | 2.26 | 2.29 | -0.03 |
| 40 | Shared interest | Impact | 2.26 | 2.26 | 2.25 | 0.01 |
| 35 | Comforted others | Impact | 2.25 | 2.23 | 2.31 | -0.08 |
| 34 | Used gestures to communicate | Impact | 2.24 | 2.24 | 2.25 | -0.01 |
| 13 | Attached to objects | Impact | 2.23 | 2.28 | 2.12 | 0.16 |
| 10 | Repeated actions | Impact | 2.21 | 2.24 | 2.13 | 0.11 |
| 37 | Used imagination | Impact | 2.21 | 2.19 | 2.25 | -0.06 |
| 21 | Had difficulty with affection | Frequency | 2.20 | 2.21 | 2.18 | 0.03 |
| 25 | Exhibited echoed speech | Impact | 2.19 | 2.23 | 2.11 | 0.12 |
| 12 | Exhibited repetitive hand and finger movements | Impact | 2.10 | 2.11 | 2.08 | 0.03 |
| 1 | Fascination with parts | Impact | 2.07 | 2.12 | 1.96 | 0.16 |
| 11 | Collected things | Impact | 2.07 | 2.10 | 2.02 | 0.08 |
| 17 | Exhibited repetitive whole body movements | Impact | 2.06 | 2.08 | 2.03 | 0.05 |
| 21 | Had difficulty with affection | Impact | 1.93 | 1.94 | 1.91 | 0.03 |
| 27* | Used a made-up or private language? | Frequency | 1.82 | 1.83 | 1.79 | 0.04 |
| 5* | Used hand over hand | Frequency | 1.77 | 1.80 | 1.71 | 0.09 |
| 27* | Used a made-up or private language? | Impact | 1.70 | 1.71 | 1.67 | 0.04 |
| 3* | Lined things up | Impact | 1.64 | 1.67 | 1.58 | 0.09 |
| 5* | Used hand over hand | Impact | 1.55 | 1.58 | 1.48 | 0.10 |

Frequency scores for items 28-41 have been reversed scored, as per AIM manual.

* These items showed a ‘floor’ effect, defined as median = 1. No items showed a ceiling effect

Table S2: Estimates for Clinically Important Responses of the AIM scores, overall and by age and IQ group (raw score scale)

| AIM | n | Total | Frequency | Impact | Repetitive  Behavior | Communi-cation | Atypical  Behavior | Social  Reciprocity | Peer Interaction |
| --- | --- | --- | --- | --- | --- | --- | --- | --- | --- |
| Overall | 4415 | 10.8 - 27.1 | 5.3 - 13.2 | 6.1 - 15.3 | 2.7 - 6.9 | 2.4 - 6.0 | 2.0 - 5.0 | 1.5 - 3.7 | 1.4 - 3.6 |
| 3-4 years | 618 | 10.0 - 24.9 | 4.8 - 11.9 | 5.9 - 14.7 | 2.6 - 6.5 | 2.1 - 5.2 | 2.0 - 4.9 | 1.5 - 3.6 | 1.4 - 3.6 |
| 5-9 years | 1903 | 10.9 - 27.3 | 5.3 - 13.3 | 6.2 - 15.5 | 2.7 - 6.8 | 2.3 - 5.8 | 2.0 - 5.0 | 1.5 - 3.8 | 1.4 - 3.6 |
| 10-14 years | 1396 | 10.7 - 26.6 | 5.3 - 13.2 | 5.9 - 14.9 | 2.7 - 6.9 | 2.2 - 5.4 | 2.0 - 5.0 | 1.4 - 3.5 | 1.4 - 3.5 |
| 15-17 years | 491 | 11.2 – 28.0 | 5.4 - 13.4 | 6.4 - 16.1 | 2.8 - 7.1 | 2.2 - 5.4 | 2.1 - 5.4 | 1.4 - 3.6 | 1.4 - 3.5 |
| IQ <70 | 390 | 10.8 - 27.1 | 5.1 - 12.7 | 6.4 – 16.0 | 2.8 – 7.0 | 2.2 - 5.6 | 2.1 - 5.3 | 1.4 - 3.4 | 1.4 - 3.5 |
| IQ 71 - 99 | 489 | 9.4 - 23.6 | 4.6 - 11.5 | 5.4 - 13.5 | 2.6 - 6.4 | 1.8 - 4.5 | 1.8 - 4.5 | 1.4 - 3.4 | 1.3 - 3.3 |
| IQ >100 | 670 | 9.6 - 23.9 | 4.7 - 11.8 | 5.4 - 13.5 | 2.6 - 6.5 | 1.5 - 3.7 | 1.9 - 4.8 | 1.3 - 3.3 | 1.3 - 3.3 |

Estimates for CIR are 0.2 - 0.5 times standard deviation. Participants with missing age or IQ data were excluded from respective analyses.

Table S3: Convergent Validity (Pearson’s correlations) between AIM Domains and SCQ and RBS-R Domains (restricted to individuals with SCQ/RBS-R performed at same age as AIM)

| AIM | SCQ (n=1,269) | | | | RBS-R (n=2,298) | | | |
| --- | --- | --- | --- | --- | --- | --- | --- | --- |
|  | Reciprocal Social Interaction | Communication | Repetition/  Stereotyped Behavior | Total | Stereotypy Restricted | Self-injurious | Compulsive, Ritualistic, Sameness | Total |
| AIM domain-Repetitive Behavior | 0.37 | 0.27 | 0.50* | 0.48 | 0.74* | 0.46 | 0.66* | 0.74 |
| AIM domain-Communication | 0.38 | 0.18* | 0.17 | 0.34 | 0.43 | 0.29 | 0.23 | 0.33 |
| AIM domain-Atypical Behavior | 0.42 | 0.31 | 0.36* | 0.48 | 0.59* | 0.43 | 0.54* | 0.59 |
| AIM domain-Social Reciprocity | 0.52* | 0.34 | 0.18 | 0.48 | 0.30 | 0.24 | 0.24 | 0.29 |
| AIM domain-Peer Interaction | 0.47* | 0.28 | 0.18 | 0.43 | 0.30 | 0.25 | 0.25 | 0.30 |
| AIM Frequency |  |  |  | 0.62 |  |  |  | 0.58 |
| AIM Impact |  |  |  | 0.48 |  |  |  | 0.62 |
| AIM Total |  |  |  | 0.57 |  |  |  | 0.63 |

* Domain correlations with pre-specified expected highest correlations of > 0.5

Exact date of SCQ/RBS-R unknown so analysis population restricted to where the child’s age (in years) at time of SCQ/RBS-R is the same as age at time of AIM. Total SCQ and total RBS-R were expected to have correlations > 0.3 with all AIM domains and summary scores

Figure S1: Mean AIM Frequency Score by Known-groups

TD: typically developing peers, rx: prescription, SLT: Speech and language therapy in last 12 months, verbal/non-verbal ability as assessed by item 1 of SCQ.

Significant differences observed across all known-groups (p<0.01 in both crude analysis and analyses adjusted for age), except for mental comorbidity vs. no mental comorbidity (p=0.41, adjusteed p=0.42). Higher scores represent higher symptom burden.

Figure S2: Mean AIM Impact Score by Known-groups

TD: typically developing peers, rx: prescription, SLT: Speech and language therapy in last 12 months, verbal/non-verbal ability as assessed by item 1 of SCQ.

Significant differences observed across all known-groups (p<0.01 in both crude analysis and analyses adjusted for age). Higher scores represent higher symptom burden.

Figure S3: Mean AIM Repetitive Behavior Domain score by Known-groups

TD: typically developing peers, rx: prescription, SLT: Speech and language therapy in last 12 months, verbal/non-verbal ability as assessed by item 1 of SCQ.

Significant differences observed across all known-groups (p<0.01 in both crude analysis and analyses adjusted for age), except for High income Medicaid vs other (p=0.77, adjusted p=0.77). Higher scores represent higher symptom burden.

Figure S4: Mean AIM Atypical Behavior Domain score by Known-groups

TD: typically developing peers, rx: prescription, SLT: Speech and language therapy in last 12 months, verbal/non-verbal ability as assessed by item 1 of SCQ.

Significant differences observed across all known-groups (p<0.01 in both crude analysis and analyses adjusted for age), except for SLT vs no SLT (p=0.17, adjusted p=0.16) and High income Medicaid vs other (p=0.40, adjusted p=0.40). Higher scores represent higher symptom burden.

Figure S5: Mean AIM Social Reciprocity Domain score by Known-groups

TD: typically developing peers, rx: prescription, SLT: Speech and language therapy in last 12 months, verbal/non-verbal ability as assessed by item 1 of SCQ.

Significant differences observed across all known-groups (p<0.01 in both crude analysis and analyses adjusted for age), except for mental comorbidity vs. no mental comorbidity (p=0.97, adjusteed p=0.98).

Figure S6: Mean AIM Peer Interaction Domain score by Known-groups

TD: typically developing peers, rx: prescription, SLT: Speech and language therapy in last 12 months, verbal/non-verbal ability as assessed by item 1 of SCQ.

Significant differences observed across all known-groups (p<0.01 in both crude analysis and analyses adjusted for age).
